# Knacks of marine predator heuristics for distributed energy source-based power systems harmonics estimation

**DOI:** 10.1016/j.heliyon.2024.e35776

**Published:** 2024-08-03

**Authors:** Khalid Mehmood Cheema, Khizer Mehmood, Naveed Ishtiaq Chaudhary, Zeshan Aslam Khan, Muhammad Asif Zahoor Raja, Ahmed M. El-Sherbeeny, Ahmed Nadeem, Zaki Ud din

**Affiliations:** a Department of Electronic Engineering, Fatima Jinnah Women University, Rawalpindi 46000, Pakistan; b Department of Electrical and Computer Engineering, International Islamic University, Islamabad, Pakistan; c Future Technology Research Center, National Yunlin University of Science and Technology, 123 University Road, Section 3, Douliou, Yunlin 64002, Taiwan; d International Graduate Institute of Artificial Intelligence, National Yunlin University of Science and Technology, 123 University Road, Section 3, Douliou, Yunlin, 64002, Taiwan; e Industrial Engineering Department, College of Engineering, King Saud University, Riyadh 11451, Saudi Arabia; f Department of Pharmacology and Toxicology, College of Pharmacy, King Saud University, Riyadh, 11451, Saudi Arabia; g Department of Engineering, Lancaster University, LA1 4YR, United Kingdom

**Keywords:** Power systems, Renewable energy systems, Marine predator algorithm, Harmonic estimation

## Abstract

The power system incorporates renewable energy resources into the main utility grid, which possesses low or no inertia, and these systems generate harmonics due to the utilization of power electronic equipment. The precise and effective assessment of harmonic characteristics is necessary for maintaining power quality in distributed power systems. In this paper, the Marine Predator Algorithm (MPA) that mimics the hunting behavior of predators is exploited for harmonics estimation. The MPA utilizes the concepts of Levy and Brownian motions to replicate the movement of predators as they search for prey. The identification model for parameter estimation of harmonics is presented, and an objective function is developed that minimizes the difference between the real and predicted harmonic signals. The efficacy of the MPA is assessed for different levels of noise, population sizes, and iterations. Further, the comparison of the MPA is conducted with a recent metaheuristic of the Reptile Search Algorithm (RSA). The statistical analyses through sufficient autonomous executions established the accurate, stable, reliable and robust behavior of MPA for all variations. The substantial enhancement in estimation accuracy indicates that MPA holds great potential as a strategy for estimating harmonic parameters in distributed power systems.

## 1 Introduction

The integration of renewable energy sources based on distributed power generator units into conventional power systems has increased rapidly over the last few years [1]. The multiple distributed power generation units develop a microgrid, and its integration with the conventional grid is possible with various sophisticated electronic power equipment [2,3]. This equipment generates various harmonics in power systems, which causes instability [4]. Further adverse effects of harmonics on power systems include overheating, resonance, interference, equipment malfunction, energy losses, etc. These factors can reduce the lifetime of the equipment and eventually lead to failure. Therefore, accurate parameter estimation of harmonics is necessary to minimize the severe effects of harmonics on the power system. Numerous researchers have proposed various techniques and methods to estimate the harmonics parameters. For example, in Ref. [5], authors proposed a technique based on partial least squares regression, which only estimates the amplitude of harmonic current and voltage. In Ref. [6], for harmonic parameter estimation, the author proposed a Slepian and Nuttall mutual convolution window, which uses the faster sidelobe and smaller sidelobe levels for precise estimation. Additionally, in Ref. [7], a frequency-adaptive Luenberger-sliding mode observer is proposed by utilizing the Lyapunov stability theorem based on frequency adaptation. The proposed frequency method is robust against several disturbances that occur in practical settings, such as irregular shifts in amplitude, frequency and phase. In Ref. [8], the authors proposed a time-domain and online harmonic parameter estimation, which is based on algebraic and asymptotic algebraic estimation methods and vibrating signal modeling. Similarly, in Ref. [9], the authors proposed a finite time parameter estimation for the online monitoring of transformers in a power system.

**Fig. 1:**
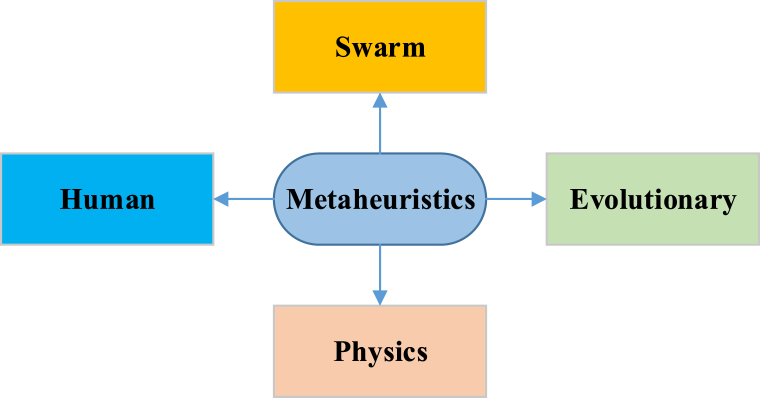
Classifications of metaheuristics.

Moreover, the metaheuristics can be divided into four major groups, as shown in Fig. 1. The first group is physics-based metaheuristics techniques, which include Memetic algorithms [10], Artificial Physics Optimization Algorithms [11], Big Bang Big Crunch [12], and Quantum-inspired evolutionary algorithms [13]. The second group is the Human-based methods, which include Driving Training-Based Optimization [14], teaching learning-based optimization [15], harmony search [16], firework algorithm [17], gaining-sharing knowledge-based algorithm [18] and society and civilization algorithm [19]. The third group includes the swarm-based methods, which include the Ant Colony Optimization [20], Particle Swarm Optimization [21], Wasp Swarm Algorithm [22], Grey Wolf Optimization [23], Firefly Algorithm [24], Fruit fly Optimization Algorithm [25], Whale optimization algorithm [26].

Subsequently, the efficiency of numerous swarm and evolutionary optimization algorithms are prevalent in the literature for harmonic parameter estimation and optimization [[27], [28], [29]]. The researchers utilize numerous optimization algorithms to estimate the harmonic parameters accurately, such as [30] incorporate the artificial bee colony mechanism [31], incorporate the sparse Bayesian learning technique [32], utilize the discrete wavelet packet transform technique [33], exploit the generalized pattern search algorithm [34], employed the backtracking search technique [35], exploited the differential evolution and [36] applied the Corrected Interpolated Discrete Fourier transform algorithm.

Comparatively, a new optimization algorithm, the marine predator algorithm, was proposed recently, which mimics the hunting behavior of marines. The MPA is applied to numerous domains, such as wireless sensor networks [37,38], 5G networks [39], electric power distribution systems [40], optimal power dispatch [41,42], image processing [43], robotics, internet of things (IoT) [44], photovoltaics [[45], [46], [47]], antennas [48,49], solar cell [50,51], prediction of COVID-19 cases [52,53], maximum power point tracking [54], due to its effective and better exploration and exploitation characteristics.

Consequently, the current research is focused on exploiting the characteristics of MPA for accurate and effective harmonic parameter estimation of microgrid-based power systems. The proposed optimization algorithm is evaluated and validated for reducing the error, and it is compared with the reptile search algorithm (RSA) [55]. The simulation analysis is carried out for various noise level variations, population sizes and iterations. Moreover, the reliability of MPA is confirmed by performing multiple tests with numerous runs. In addition, sub and interharmonic analysis is performed to endorse the proposed methodology. The main contributions of the study are.

• Design of MPA heuristics for accurate and robust parameter estimation of harmonics as well as inter/subharmonics scenarios in power systems.
• The MPA incorporates the concepts of Levy and Brownian motions to replicate the movement of predators as they search for prey.
• The mean performance behavior based on sufficient autonomous executions confirms the stability and reliability of the MPA for harmonics estimation.
• Comparing the MPA with its recent counterpart endorses the scheme's efficacy for different populations, iterations and noise scenarios.
• The substantial enhancement in estimation accuracy indicates that MPA holds great potential as a strategy for estimating harmonic parameters in distributed power systems.

Besides these, the rest of the paper is organized as follows. Section 2 discussed the proposed methodology of the harmonic identification model. Section 3 discusses the MPA optimization heuristic. Section 4 discuss the performance analysis of simulation results. Section 5 presents sub and interharmonic analysis. Section 6 presents the conclusion of the current research work.

## 2 Harmonics identification model

Mathematically, the electrical harmonic signal, in terms of the amplitude, frequency and phase parameters signal, can be written as [13,14]:

(1) $$x\left(t\right)=\sum_{n}^{N}\sigma_{n}\;\sin\left(\omega_{n}t+\lambda_{n}\right)+\Delta \left(t\right)\text{,}$$

where ω_*n*_ represents the angular frequency of the nth harmonic, and it is defined as $\omega_{n}=2n\pi f$ while f depicts the fundamental frequency. *λ*_*n*_ and σ_*n*_ are the phase and amplitude corresponding to the nth harmonic. *Δ* depicts the additive white Gaussian noise, and N represents the harmonic order. Rewrite Equation (1) in discreet form by modifying the signal *x*(*t*) with period *l*, then *t*_*p*_ = *pl*

(2) $$x\left(t_{p}\right)=\sum_{n}^{N}\sigma_{n}\;\sin\left(\omega_{n}t_{p}+\lambda_{n}\right)+\Delta \left(t_{p}\right)\text{.}$$

To avoid complications, let assume *s*(*t*_*p*_) = *s*(*p*) and modify Equation (2) as Equation (3):

(3) $$x\left(p\right)=\sum_{n}^{N}\sigma_{n}\;\sin\left(\omega_{n}p+\lambda_{n}\right)+\Delta \left(p\right)\text{.}$$

Now apply the fundamental trigonometric identity to Equation (2) and present it in terms of sine and cosine forms as Equation 4

(4) $$x\left(p\right)=\sum_{n=1}^{N}\left(\sigma_{n}\;\sin\left(\omega_{n}p\right)\cos\;\lambda_{n}+\sigma_{n}\;\cos\left(\omega_{n}p\right)\sin\;\lambda_{n}\right)+\Delta \left(p\right)\text{.}$$

Let assume $\sigma_{n}\;\cos\;\lambda_{n}=y_{n}$ and $\sigma_{n}\;\sin\;\lambda_{n}=z_{n}$, now we can rewrite Equation (4) as Equation 5

(5) $$x\left(p\right)=\sum_{n=1}^{N}\left(y_{n}\;\sin\left(\omega_{n}p\right)+z_{n}\;\cos\left(\omega_{n}p\right)\right)+\Delta \left(p\right)\text{.}$$

For the identification model, we can write Equation (5) as Equation (6) and Equation 7

(6) $$x\left(p\right)=q^{T}\left(p\right)r+\Delta \left(p\right)\text{,}$$

where

(7) $$q\left(p\right)=\left(\sin\left(\omega_{1}p\right),\cos\left(\omega_{1}p\right),\sin\left(\omega_{2}p\right),\cos\left(\omega_{2}p\right),....,\sin\left(\omega_{n}p\right),\cos\left(\omega_{n}p\right)\right)\text{,}$$

and

(8) $$r=\left(y_{1},z_{1},y_{2},z_{2},....,y_{n},z_{n}\right)\text{.}$$

The main objective of this research is the estimation of parameters of distributed power system harmonics by reducing the difference between actual harmonic x(p) and estimated harmonic $\hat{x}\left(p\right)$ by utilizing the MPA. Therefore, the objective function can be expressed as

(9) $$\alpha \left(p\right)=mean{\left(x\left(p\right)-\hat{x}\left(p\right)\right)}^{2}={\left(x\left(p\right)-q^{T}\left(p\right)\hat{r}\right)}^{2}\text{.}$$

As can be observed, the identification model is mentioned in Equation (6), and the cost function is mentioned in Equation (9). Therefore, the intermediate variable can be considered as a parameter that needs to be identified. Moreover, it is essential to use related terms for Equation (8) and Equation (3). Therefore, the essential relations are given as Equation 10

(10) $$\sigma_{n}=\sqrt{{\left(y_{n}\right)}^{2}+{\left(z_{n}\right)}^{2}},\lambda_{n}=\tan^{-1}\frac{z_{n}}{y_{n}}\text{.}$$

## 3 Methodology

**Fig. 2:**
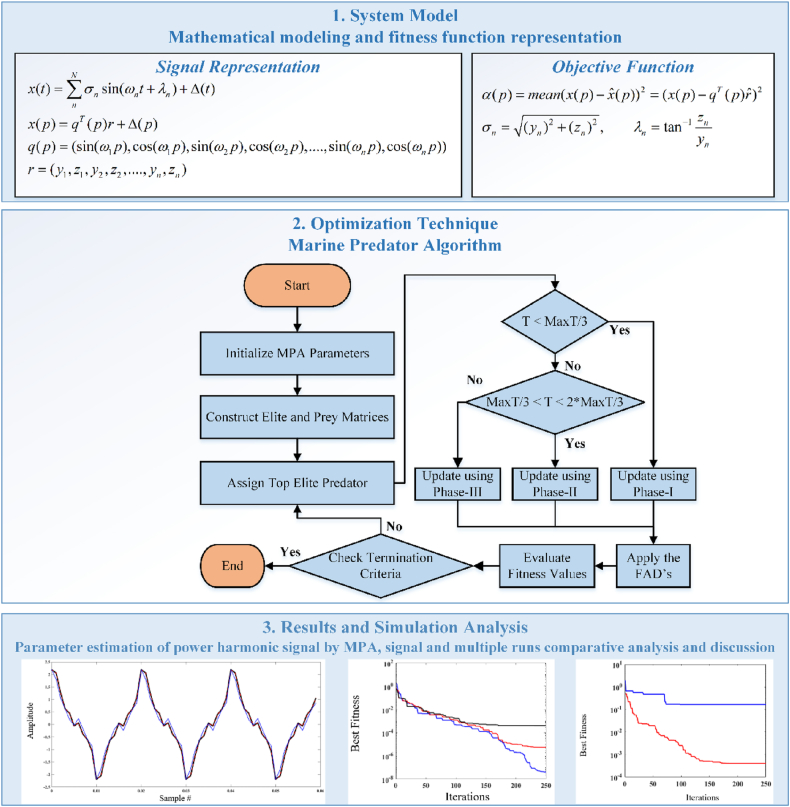
Graphical abstract of proposed methodology.

The methodology for the marine predator algorithm (MPA) for the harmonic parameter identification model is explained in detail according to the mathematical development and process flow depiction. A simplified and general overview of the considering the fundamental blocks is shown in Fig. 2.

The MPA is a metaheuristic based on populations of marine predators to catch prey [56]. The MPA uses Levy and Brownian motion to make the best contact between prey and predator. Considering the mathematical modeling, the MPA population is distributed uniformly all over the search space, and it can be stated as Equation (11):

(11) $$P_{0}=P_{\min}+rand\left(P_{\max}-P_{\min}\right)\text{,}$$

where *P*_max_ and *P*_min_ represent the maximum and minimum constraints for parameters. Moreover, rand represents the uniform accidental vector with a range between (0–1). Considering the theory of “survival of the fittest”, the predator with extra skills is the best. Thus, such a predator is the appropriate solution to create a matric, which can be called an Elite matrix, and it can be expressed as Equation (12):

(12) $$E={\left(\begin{array}{ccc} B_{1,1}^{I} & \ldots & B_{1,i}^{I}\\ \vdots & \ddots & \vdots \\ B_{j,1}^{I} & \cdots & B_{j,i}^{I} \end{array}\right)}_{j\times i}\text{,}$$

where *i* represents the dimensions, *j* represents the simulation runs, and *B*^*I*^ represents the best predator. Similarly, the prey matrix can be expressed as Equation (13).

(13) $$P={\left(\begin{array}{ccc} B_{1,1} & \ldots & B_{1,i}\\ \vdots & \ddots & \vdots \\ B_{j,1} & \cdots & B_{j,i} \end{array}\right)}_{j\times i}\text{.}$$

Considering the speed of prey and predator, the MPA can be divided into three phases. The details of these sections are mentioned below.

### 3.1 Phase 1

According to the first phase, by realizing the Brownian movement, it is assumed that the predator speed is higher than the prey speed for the initial 1/3 iterations. Mathematically, the prey matrix can be expressed as Equation (14) and Equation (15):

(14) $${\vec{S}}_{k}={\vec{R}}_{B}⊗\left({\vec{E}}_{k}-\left({\vec{R}}_{B}⊗{\vec{P}}_{k}\right)\right),k=1,2,....j\text{,}$$

(15) $${\vec{P}}_{k}={\vec{P}}_{k}+\left(F\times \vec{R}⊗{\vec{S}}_{k}\right)\text{,}$$

where ${\vec{S}}_{k}$ denotes the step size, F represents a fixed value, which is 0.5, and $\vec{R}$ denotes the vector € [0.1], and ${\vec{R}}_{B}$ denotes the accidental amount during the Brownian movement.

### 3.2 Phase 2

In this phase, it is assumed that the predator and prey both move at the same speed and both are searching for their prey. Moreover, the predator movement is under Brownian motion, and the prey movement is under Levy motion from 1/3 to 2/3 iterations. Considering these assumptions, the matrix can be updated as Equation (16), (17), (18), (19)):

(16) $${\vec{S}}_{k}={\vec{R}}_{L}⊗\left({\vec{E}}_{k}-\left({\vec{R}}_{L}⊗{\vec{P}}_{k}\right)\right),k=1,2,....\frac{j}{2}\text{,}$$

(17) $${\vec{P}}_{k}={\vec{P}}_{k}+\left(F\times \vec{R}⊗{\vec{S}}_{k}\right)\text{,}$$

(18) $${\vec{S}}_{k}={\vec{R}}_{B}⊗\left({\vec{R}}_{B}⊗\left({\vec{E}}_{k}-⊗{\vec{P}}_{k}\right)\right),k=1,2,....j\text{,}$$

(19) $${\vec{P}}_{k}={\vec{E}}_{k}+\left(F\times AD⊗{\vec{S}}_{k}\right)\text{,}$$

where R_L_ represents the accidental amount during the Levy movement. The AD represents the defining factor for controlling the predator movement step size, and it can be expressed as Equation (20):

(20) $$AD={\left(1-\frac{itera}{\max_{itera}}\right)}^{\left(\frac{2itera}{\max_{itera}}\right)}$$

### 3.3 Phase 3

It is assumed that the predator moves at a higher speed than its prey. Therefore, the Levy movement is applied to the predator movement, and the matrix can be represented as Equation (21) and Equation (22):

(21) $${\vec{S}}_{k}={\vec{R}}_{L}⊗\left({\vec{R}}_{L}⊗\left({\vec{E}}_{k}-⊗{\vec{P}}_{k}\right)\right),k=1,2,....j\text{,}$$

(22) $${\vec{P}}_{k}={\vec{E}}_{k}+\left(F\times AD⊗{\vec{S}}_{k}\right)\text{.}$$

**Fig. 3:**
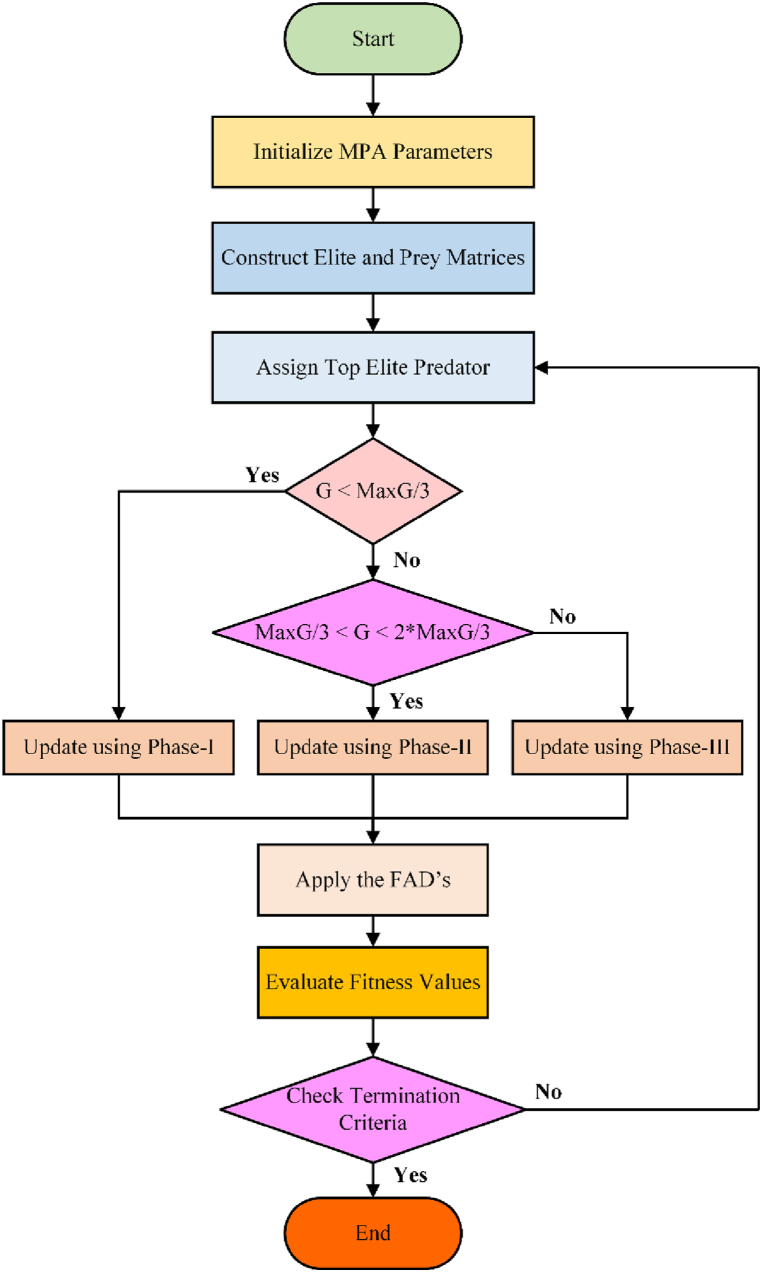
Flowchart of MPA

Besides these three sections, the effects of the Fish Aggregating Devices (FADs) are incorporated into MPA to mimic natural marine behaviour. According to FADs, the fish spend more than 80 % of their time near the FAD and for less than 20 %, fish jump around in different locations away from prey, and it can be expressed as Equation (23):

(23) $${\vec{P}}_{k}=\left\{ \begin{array}{c} {\vec{P}}_{k}+AD\left[P_{\min}+\vec{R}⊗\left(P_{\max}-P_{\min}\right)\right]⊗\vec{V}\;if\;z\leq FAD\\ {\vec{P}}_{k}+\left[FAD\left(1-z\right)+z\right]\left(P_{z1}-P_{z2}\right)\;if\;z>FAD \end{array}\right.\text{,}$$

where $\vec{V}$ represents a binary vector, z depicts that it is a random number ∈[0.1], z_1_ and z_2_ represent that they are the subscripts of the prey matrix, and FAD = 0.2. Furthermore, the MPA working process is presented in Fig. 3.

## 4 Performance analysis

The performance of MPA and RSA is evaluated for iterations T (125, 250, 375), populations P (20, 40) and noise n (0.0002, 0.002, 0.02). The evaluation of the algorithms is carried out by considering the accuracy and robustness of the fitness function mentioned in Equation 24

(24) $$Fitness\;Function=mean{\left(x-\hat{x}\right)}^{2}\text{,}$$

where *x* and $\hat{x}$ are the required and estimated responses, respectively. Moreover, the distributed power systems' harmonic signal is taken from the [30,57] and mentioned as Equation 25

(25) $$x=1.5*\sin\left(2*\pi *f_{1}*t+1.396\right)+0.5*\sin\left(2*\pi *f_{3}*t+1.047\right)+0.2*\sin\left(2*\pi *f_{5}*t+0.785\right)+0.15*\sin\left(2*\pi *f_{7}*t+0.628\right)+0.1*\sin\left(2*\pi *f_{11}*t+0.523\right)\text{.}$$

Additionally, the estimated parameters of the distributed power system are as Equation 26

(26) $$\left[\begin{array}{c} \beta_{1},\beta_{2},\beta_{3},\beta_{4},\beta_{5}\\ \gamma_{1},\gamma_{2},\gamma_{3},\gamma_{4},\gamma_{5} \end{array}\right]=\left[\begin{array}{c} 1.50,0.50,0.20,0.15,0.10\\ 1.396,1.047,0.785,0.628,0.523 \end{array}\right]$$

**Fig. 4:**
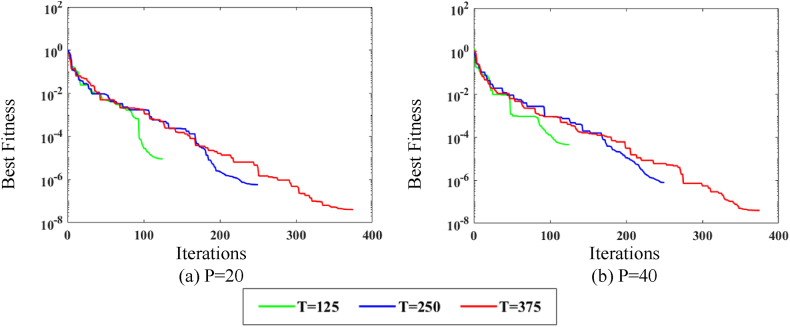
MPA fitness curves w.r.t iterations.

The convergence curve depicted in Fig. 4 illustrates the behavior of P (20,40) over three T (125, 250, 375). Fig. 4(a) corresponds to P = 20 over all iterations, while Fig. 4(b) corresponds to P = 40 over all iterations. The noise level is set to a minimum for this analysis. It is observed in Fig. 4(a) and (b) that when the P size increases, the fitness value improves significantly for all variations of T.

**Fig. 5:**
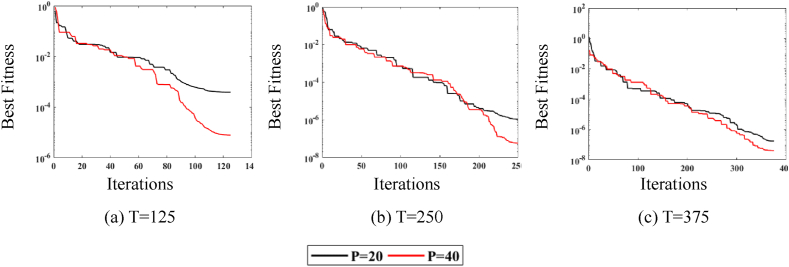
MPA fitness curves w.r.t population sizes.

Consequently, the convergence curve depicted in Fig. 5 illustrates the behavior of T (125, 250, 375) for both values of P (20,40). Fig. 5(a) corresponds to T = 125 over both populations, while Fig. 5(b) and (c) represent the same scenario for T = 250 and T = 375, respectively. It is observed in Fig. 5(a)–5(c) that when the T increases, the fitness value improves significantly for both values of P.

**Fig. 6:**
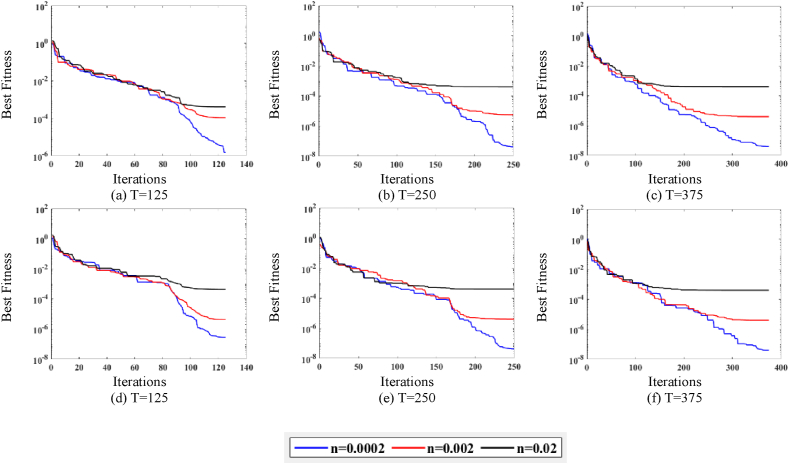
MPA fitness curves w.r.t noise levels.

Fig. 6 illustrates the behavior of MPA. The P is set as (20,40), and the T is set as (125, 250, 375), whereas the noise levels are set as n = (0.0002, 0.002, 0.02). Fig. 6(a)–(c) depicts the behavior of noises at P = (20), while Fig. 6(d)–(f) shows the behavior of noises at P = (40). Moreover, it is observed in Fig. 6(a)–(f) that for the fixed value of P and T, the fitness attained by MPA is low for the low level of noise and fairly high for the higher level of noise. Consequently, it is concluded that the MPA performance degraded significantly due to the higher noise values.

The performance of MPA is evaluated for three levels of n = (0.0002, 0.002, 0.02), and these are presented in Table 1, Table 2, Table 3, respectively. It is observed in Table 1, Table 2, Table 3 data that by increasing the values of P and T, the fitness of MPA improves considerably. Considering Table 1, for n = 0.0002, the average fitness, best fitness and worst fitness values are 4.1343e-08, 4.0280e-08 and 4.9154e-08, respectively. Likewise, for n = 0.002, the average fitness, best fitness and worst fitness values are 4.0259e-06, 4.0252e-06 and 4.0301e-06, respectively. Additionally, for n = 0.02, the average fitness, best fitness and worst fitness values are 4.0251e-04, 4.0250e-04 and 4.0252e-04, respectively. Furthermore, it is observed that the values of average, best and worst fitness degraded with the increment of noise level.

**Table 1:** MPA analysis with respect to iteration and population sizes at 0.0002 noise level.

T	P	Average	Best	Worst
125	20	9.4084e-05	8.8447e-07	4.7718e-04
	40	3.3346e-05	6.4328e-08	2.1019e-04
250	20	1.8388e-07	4.1621e-08	1.7247e-06
	40	8.4412e-08	4.0520e-08	7.9119e-07
375	20	4.5653e-08	4.0360e-08	1.7494e-07
	40	4.1343e-08	4.0280e-08	4.9154e-08

**Table 2:** MPA analysis with respect to iteration and population sizes at 0.002 noise level.

T	P	Average	Best	Worst
125	20	9.3623e-05	4.9567e-06	5.2949e-04
	40	2.2203e-05	4.0513e-06	1.2574e-04
250	20	4.2891e-06	4.0272e-06	7.5637e-06
	40	4.1119e-06	4.0254e-06	6.1456e-06
375	20	4.0283e-06	4.0254e-06	4.0532e-06
	40	4.0259e-06	4.0252e-06	4.0301e-06

**Table 3:** MPA analysis with respect to iteration and population sizes at 0.02 noise level.

T	P	Average	Best	Worst
125	20	4.6051e-04	4.0277e-04	6.6953e-04
	40	4.3855e-04	4.0265e-04	7.6708e-04
250	20	4.0290e-04	4.0252e-04	4.1103e-04
	40	4.0257e-04	4.0251e-04	4.0304e-04
375	20	4.0252e-04	4.0251e-04	4.0269e-04
	40	4.0251e-04	4.0250e-04	4.0252e-04

**Fig. 7:**
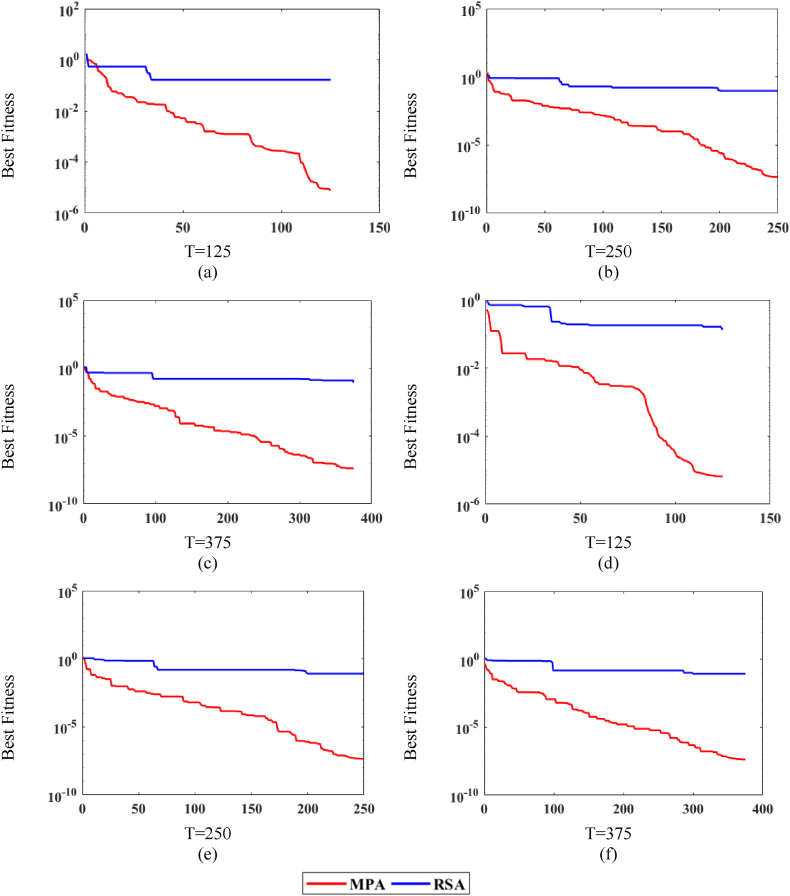
MPA and RSA convergence curves at n = 0.0002.

**Fig. 8:**
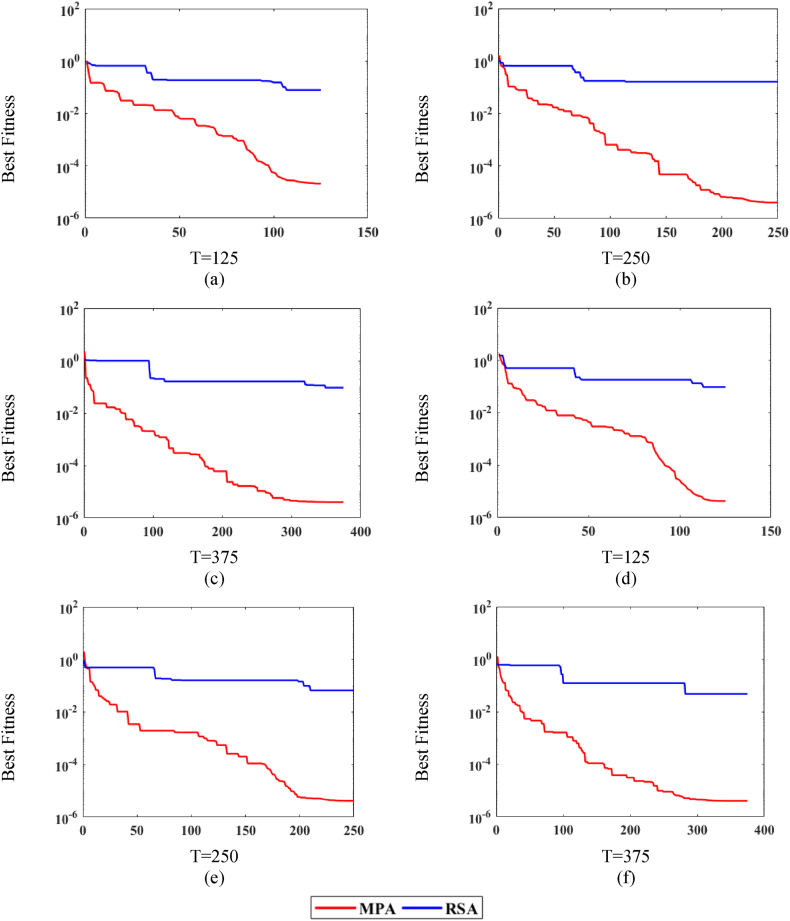
MPA and RSA convergence curves at n = 0.002.

**Fig. 9:**
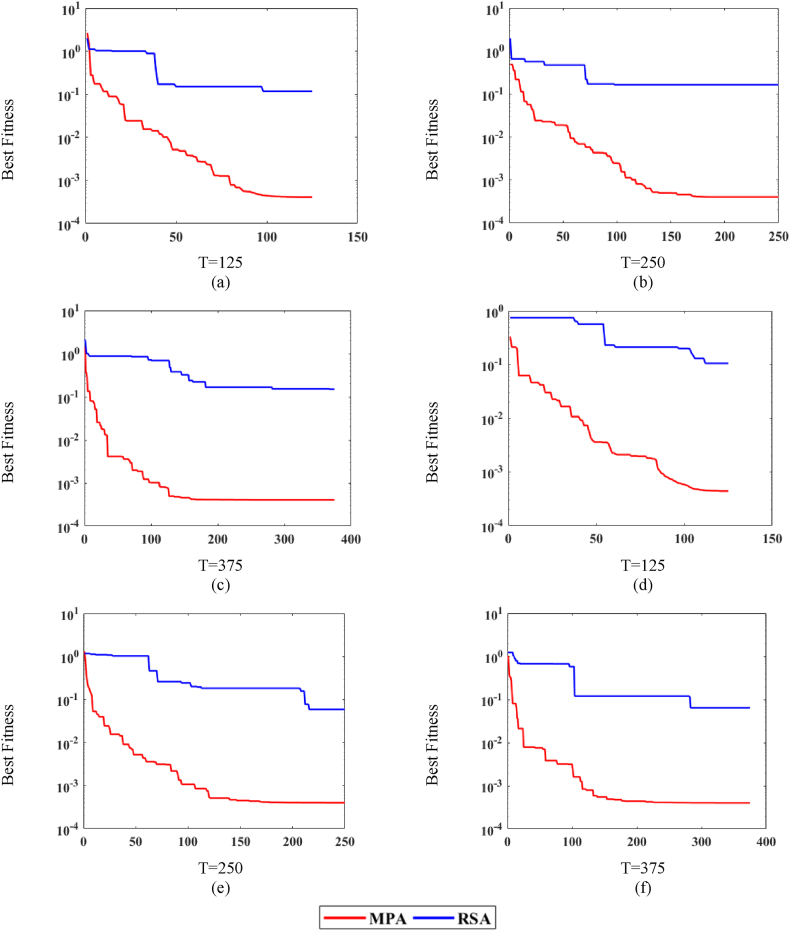
MPA and RSA convergence curves at n = 0.02.

Fig. 7, Fig. 8, Fig. 9 shows the comparison of MPA with RSA on all variations of noise levels, P and T. Fig. 7 (a) – 7 (c) shows the convergence curves at n = 0.0002 for P = 20, whereas Fig. 7 (d) – 7 (f) shows the curves for P = 40. Correspondingly, Fig. 8 (a) – 8 (c) shows the curves at n = 0.002 for P = 20, while Fig. 8 (d) – 8 (f) shows the curves for P = 40. Likewise, Fig. 9 (a) – 9 (c) shows the curves at n = 0.02 for P = 20, while Fig. 9 (d) – 9 (f) shows the curves for P = 40. It is observed from Fig. 7, Fig. 8, Fig. 9 that MPA achieves better fitness value than RSA in all variations. Moreover, it is also observed that the performance of both methods lowers at high noise levels.

**Fig. 10:**
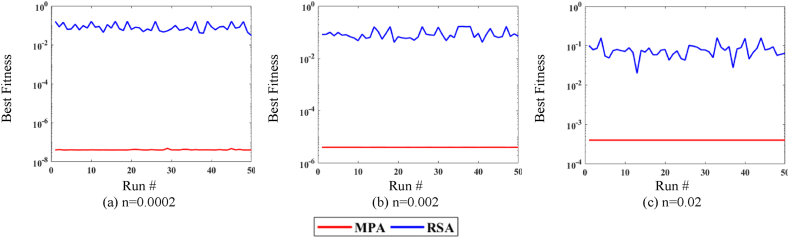
MPA and RSA statistical analyses plots for P = 40 and T = 375.

Additionally, for further exploration and validation of MPA performance, it is compared with RSA. The values of T, P, and n remain the same for the RSA algorithm and the comparison is presented in Fig. 10. It is observed in Fig. 10(a)–(c) that MPA performs significantly better than RSA for all noise levels in independent runs. Additionally, it can be observed that for the higher value of noise, the fitness increases for both algorithms.

Consequently, Table 4, Table 5, Table 6 present the optimal amplitude and phases computed by the MPA and RSA algorithm for the distributed power system. It can be seen that Table 4, Table 5, Table 6 shows the best fitness for three levels of n, and for the lowest level of n, the MPA gives the best fitness, and for the highest level of n, the best fitness degraded significantly.

**Table 4:** Comparison of MPA with RSA against best fit for the HPE model at 0.0002 noise level.

Method	G	P	Best Fitness	*β*_*1*_	*β*_*2*_	*β*_*3*_	*β*_*4*_	*β*_*5*_	*γ*_*1*_	*γ*_*2*_	*γ*_*3*_	*γ*_*4*_	*γ*_*5*_
MPA	125	20	8.8447e-07	1.5001	0.5002	0.1996	0.1498	0.1010	1.3960	1.0470	0.7869	0.6251	0.5265
		40	6.4328e-08	1.5000	0.4999	0.2001	0.1500	0.1001	1.3960	1.0470	0.7853	0.6282	0.5246
	250	20	4.1621e-08	1.5000	0.4999	0.2000	0.1500	0.1000	1.3960	1.0470	0.7850	0.6281	0.5227
		40	4.0520e-08	1.5000	0.4999	0.2000	0.1500	0.1000	1.3960	1.0470	0.7850	0.6281	0.5228
	375	20	4.0360e-08	1.5000	0.4999	0.2000	0.1500	0.1000	1.3960	1.0470	0.7850	0.6282	0.5229
		40	4.0280e-08	1.5000	0.4999	0.2000	0.1500	0.1000	1.3960	1.0470	0.7850	0.6281	0.5230
RSA	125	20	0.0284	1.4114	0.5031	0.1051	0.0536	0.0000	1.3254	0.8590	0.6165	0.9064	0.9773
		40	0.0473	1.3398	0.4313	0.1874	0.0591	0.0946	1.2828	0.7027	0.5960	0.1029	0.2071
	250	20	0.0230	1.4307	0.4594	0.0589	0.2432	0.0323	1.3466	1.0158	0.7931	0.7181	0.0535
		40	0.0370	1.5039	0.5072	0.0233	0.0608	0.0780	1.3072	0.8206	0.8455	0.1054	0.1366
	375	20	0.0233	1.5086	0.5156	0.2379	0.2227	0.1900	1.4023	1.0328	1.3576	0.9320	1.3752
		40	0.0300	1.3284	0.5473	0.1360	0.2417	0.1017	1.3756	1.1589	0.8344	0.8952	1.4935

**Table 5:** Comparison of MPA with RSA against best fit for the HPE model at 0.002 noise level.

Method	G	P	Best Fitness	*β*_*1*_	*β*_*2*_	*β*_*3*_	*β*_*4*_	*β*_*5*_	*γ*_*1*_	*γ*_*2*_	*γ*_*3*_	*γ*_*4*_	*γ*_*5*_
MPA	125	20	4.9567e-06	1.4996	0.4992	0.2008	0.1510	0.0998	1.3960	1.0473	0.7851	0.6284	0.5177
		40	4.0513e-06	1.4997	0.4993	0.2003	0.1500	0.1002	1.3960	1.0475	0.7851	0.6286	0.5215
	250	20	4.0272e-06	1.4997	0.4993	0.2002	0.1499	0.1002	1.3960	1.0472	0.7847	0.6284	0.5215
		40	4.0254e-06	1.4997	0.4993	0.2003	0.1499	0.1002	1.3960	1.0472	0.7847	0.6289	0.5222
	375	20	4.0254e-06	1.4997	0.4993	0.2003	0.1499	0.1002	1.3960	1.0472	0.7845	0.6289	0.5222
		40	4.0252e-06	1.4997	0.4993	0.2003	0.1499	0.1002	1.3960	1.0472	0.7846	0.6291	0.5220
RSA	125	20	0.0411	1.3242	0.5864	0.0277	0.1569	0.1561	1.4128	0.9220	0.2174	0.2753	0.2590
		40	0.0298	1.4945	0.5467	0.3596	0.0522	0.1115	1.3456	0.8641	0.8126	0.3220	1.3151
	250	20	0.0408	1.5171	0.5372	0.0976	0.0833	0.0000	1.3645	1.4087	0.7082	1.9138	0.1381
		40	0.0450	1.2554	0.6239	0.1703	0.0762	0.0489	1.4126	0.9580	0.6605	1.0868	0.6683
	375	20	0.0258	1.3674	0.5106	0.1959	0.1749	0.0782	1.2793	0.9585	0.6738	0.4216	0.9539
		40	0.0415	1.4628	0.3934	0.2233	0.0006	0.0681	1.2798	0.8101	0.4730	0.6711	0.0016

**Table 6:** Comparison of MPA with RSA against best fit for the HPE model at 0.02 noise level.

Method	G	P	Best Fitness	*β*_*1*_	*β*_*2*_	*β*_*3*_	*β*_*4*_	*β*_*5*_	*γ*_*1*_	*γ*_*2*_	*γ*_*3*_	*γ*_*4*_	*γ*_*5*_
MPA	125	20	4.0277e-04	1.4970	0.4929	0.2026	0.1492	0.1015	1.3964	1.0486	0.7813	0.6367	0.5112
		40	4.0265e-04	1.4969	0.4929	0.2027	0.1487	0.1022	1.3964	1.0488	0.7808	0.6384	0.5136
	250	20	4.0252e-04	1.4970	0.4929	0.2028	0.1488	0.1018	1.3964	1.0487	0.7810	0.6369	0.5145
		40	4.0251e-04	1.4969	0.4929	0.2028	0.1488	0.1018	1.3964	1.0488	0.7810	0.6372	0.5151
	375	20	4.0251e-04	1.4969	0.4929	0.2028	0.1488	0.1018	1.3964	1.0487	0.7811	0.6372	0.5152
		40	4.0251e-04	1.4969	0.4929	0.2028	0.1488	0.1018	1.3964	1.0487	0.7810	0.6372	0.5151
RSA	125	20	0.0305	1.3472	0.3796	0.1277	0.0869	0.1578	1.3612	0.9609	0.7217	0.0426	1.0189
		40	0.0260	1.4401	0.4671	0.2680	0.1006	0.1459	1.3107	0.7934	0.8465	0.2722	1.1589
	250	20	0.0263	1.4773	0.6484	0.2374	0.2688	0.0728	1.3410	1.0070	0.6439	0.7276	1.0957
		40	0.0380	1.4442	0.5261	0.0000	0.0265	0.0153	1.3663	1.0743	3.0000	1.8946	0.8664
	375	20	0.0320	1.4123	0.6008	0.2791	0.2877	0.0000	1.3821	1.0987	0.9898	0.9935	0.9478
		40	0.0198	1.4155	0.4695	0.1930	0.1128	0.0379	1.4037	0.7665	0.4301	0.2587	0.0813
Actual			0	1.5	0.5	0.2	0.15	0.1	1.396	1.047	0.785	0.628	0.523

It can be seen from Table 4, Table 5, Table 6 that the best weights are estimated for low noise levels, i.e., n = 0.0002. However, with an increase in noise levels, the fitness also increases thereby making a difference between actual and estimated value. It is also noted that for all variations of n, T and P, the MPA outperforms RSA in terms of fitness values and estimated weights.

**Fig. 11:**
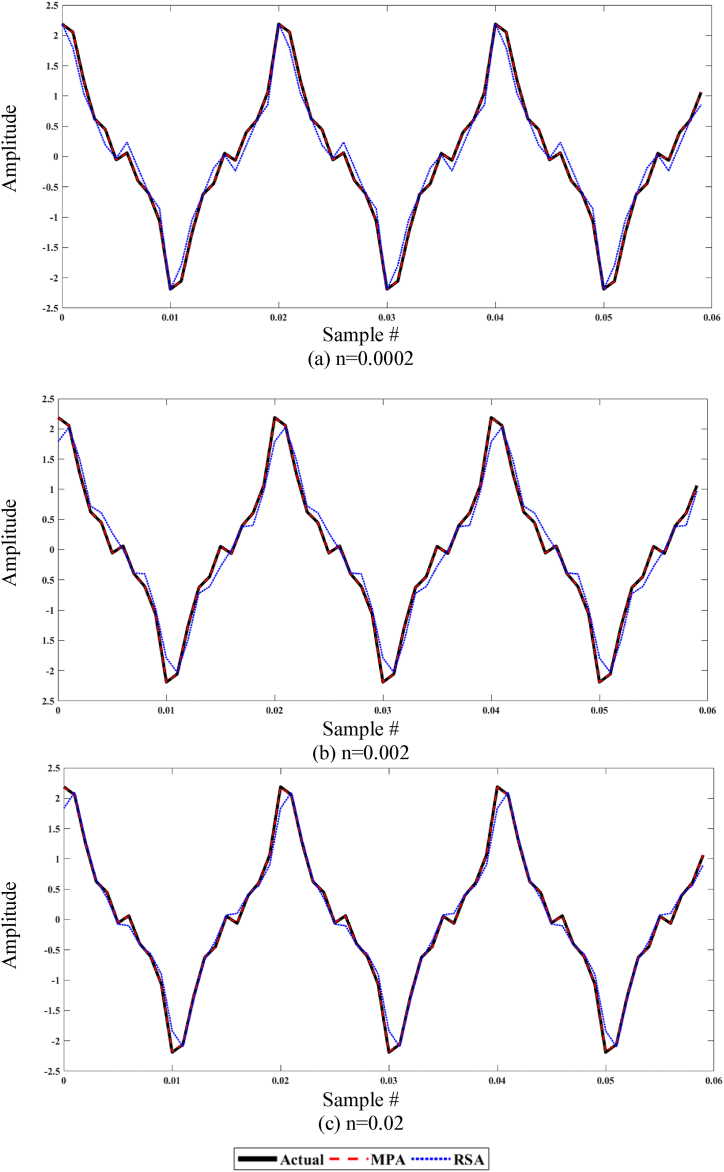
MPA and RSA curve fitting plot w.r.t actual curve for P = 40 and T = 375.

In Fig. 11, the curve fitting through MPA and RSA algorithms is shown for fix values of P and T and different values of n. Considering Fig. 11(a–c) for the all values of n, it is observed that the estimated values by MPA are fairly close to the actual signal whereas the estimated values by RSA are somewhat inaccurate in phase as well as amplitude.

## 5 Sub and interharmonic analysis

In sub and interharmonics, MPA and RSA are assessed for P = 40 and T = 375. The actual power signal is taken from Ref. [30] as mentioned in Equation (27).

(27) $$x=0.505*\sin\left(2*\pi *f_{sub}*t+75^{0}\right)+1.5*\sin\left(2*\pi *f_{1}*t+80^{0}\right)+0.5*\sin\left(2*\pi *f_{3}*t+60^{0}\right)+0.25*\sin\left(2*\pi *f_{\mathrm{int}1}*t+65^{0}\right)+0.35*\sin\left(2*\pi *f_{\mathrm{int}2}*t+20^{0}\right)+0.2*\sin\left(2*\pi *f_{5}*t+45^{0}\right)+0.15*\sin\left(2*\pi *f_{7}*t+36^{0}\right)+0.1*\sin\left(2*\pi *f_{11}*t+30^{0}\right)+0.5*\exp\left(-5*t\right)+n\text{.}$$

**Fig. 12:**
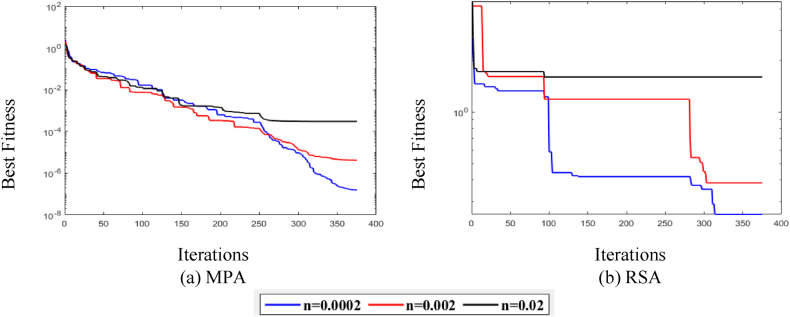
MPA and RSA fitness curves w.r.t noise levels.

Fig. 12 illustrates the behavior of MPA and RSA. The P is set as (40), and the T is set as (375), whereas the noise levels are set as n = (0.0002, 0.002, 0.02). The fitness attained by MPA and RSA is low for the low level of noise and fairly high for the higher level of noise, as illustrated in Fig. 12 (a) and 12 (b), respectively. Consequently, it is concluded that the MPA and RSA performance degraded significantly due to the higher noise values.

**Fig. 13:**
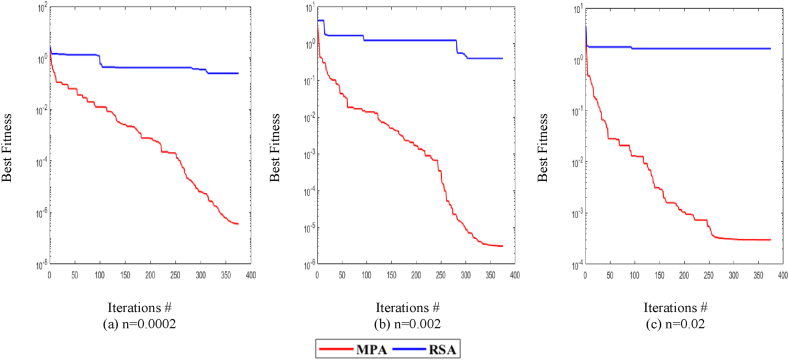
MPA and RSA interharmonic convergence analyses for P = 40 and T = 375.

Fig. 13 compares MPA with RSA for all variations of noise levels, P and T. Fig. 13(a)–(c) show that MPA attains a better fitness value than RSA for all variations.

**Fig. 14:**
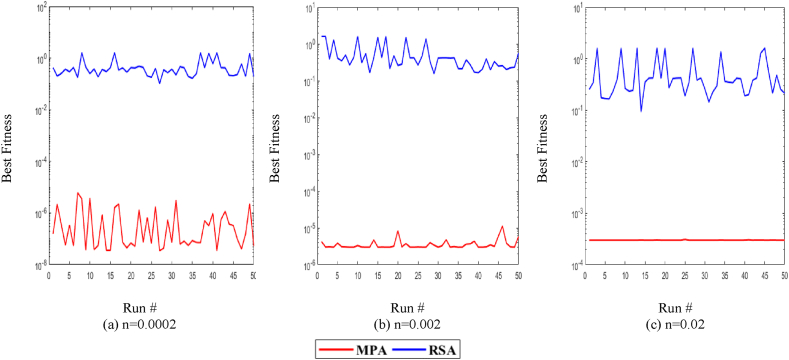
MPA and RSA interharmonic statistical analyses for P = 40 and T = 375.

For further exploration and validation of MPA performance, a statistical comparison is performed with RSA for fifty independent runs. It is observed in Fig. 14(a)–(c) that MPA performs significantly more than RSA for all noise levels in independent runs.

Table 7 presents the optimal amplitude and phases computed by the MPA and RSA algorithms for the distributed power system. It can be seen that Table 7 shows the average fitness for three levels of n, and for the lowest level of n, the MPA give the best average fitness and for the highest level of n, the average fitness degraded significantly.

**Table 7:** Comparison of MPA with RSA against average fit for the HPE model.

**Algorithm**	n	$w_{1}$	$w_{2}$	$w_{3}$	$w_{4}$	$w_{5}$	$w_{6}$	$w_{7}$	$w_{8}$	$w_{9}$	$w_{10}$	$w_{11}$	$w_{12}$	$w_{13}$	$w_{14}$	$w_{15}$	$w_{16}$	**Average Fitness**
**MPA**	0.0002	0.51	1.50	0.50	0.25	0.35	0.20	0.15	0.10	1.31	1.40	1.05	1.14	0.35	0.78	0.63	0.53	7.38E-07
	0.002	0.51	1.50	0.50	0.25	0.35	0.20	0.15	0.10	1.31	1.40	1.05	1.14	0.35	0.78	0.63	0.54	3.67E-06
	0.02	0.51	1.50	0.50	0.05	0.35	0.20	0.15	0.10	1.32	1.40	1.04	1.14	0.35	0.76	0.62	0.58	3.01E-04
**RSA**	0.0002	0.20	1.22	0.23	0.30	0.56	0.35	0.13	0.00	1.09	1.14	0.86	0.58	0.50	0.94	1.03	2.11	4.77E-01
	0.002	0.31	1.02	0.81	0.47	0.47	0.40	0.26	0.24	0.91	1.02	1.00	1.05	1.08	0.93	0.78	0.75	5.19E-01
	0.02	0.33	1.02	0.28	0.41	0.32	0.17	0.23	0.03	1.39	1.08	0.54	0.53	0.08	0.72	0.86	0.34	5.32E-01
True Weights		0.50	1.50	0.50	0.25	0.35	0.20	0.15	0.10	1.39	1.04	0.78	0.62	0.52	1.30	1.13	0.34	0

## 6 Conclusion

This study addresses the harmonics problem of modern power systems by exploiting the Marine Predator Algorithm (MPA), mimicking the hunting behavior of predators. The MPA utilizes the concepts of Levy and Brownian motions to replicate the movement of predators as they search for prey. The mathematical model for the identification of amplitude and phase parameters of harmonics is presented, and then the error-based objective function is developed to minimize the difference between the real and predicted harmonic signals. The efficacy of the MPA is deeply evaluated for different levels of noise, population sizes, and iterations. The accuracy level of the MPA is enhanced with an increase in iterations. The reliable inferences are drawn by comparing the MPA with the recent metaheuristic of the Reptile Search Algorithm (RSA) for multiple autonomous executions. The best fitness values of MPA are 4.0520e-08, 4.0254e-06 and 4.0251e-04 for noise levels 0.0002, 0.002, and 0.02, respectively. The worst fitness levels are 7.9119e-07, 6.1456e-06, and 4.0304e-04 for noise levels of 0.0002, 0.002, and 0.02, respectively. These statistical observations established the accurate, stable, reliable and robust behavior of MPA for all variations. The proposed scheme has the potential to improve power system reliability, thereby facilitating the widespread adoption of renewable energy sources and enhancing overall grid resilience in the face of increasing energy demands and environmental challenges.

## CRediT authorship contribution statement

**Khalid Mehmood Cheema:** Writing – original draft, Software, Investigation, Conceptualization. **Khizer Mehmood:** Software, Formal analysis, Data curation, Conceptualization. **Naveed Ishtiaq Chaudhary:** Validation, Project administration, Methodology, Investigation, Formal analysis. **Zeshan Aslam Khan:** Writing – review & editing, Visualization, Validation, Software, Resources. **Muhammad Asif Zahoor Raja:** Writing – review & editing, Visualization, Supervision, Resources. **Ahmed M. El-Sherbeeny:** Visualization, Supervision, Resources, Project administration. **Ahmed Nadeem:** Visualization, Supervision, Resources, Project administration, Methodology. **Zaki Ud din:** Writing – review & editing, Visualization, Validation, Software.

## Declaration of competing interest

The authors declare that they have no known competing financial interests or personal relationships that could have appeared to influence the work reported in this paper.
